# Sesquiterpene binding Gly-Leu-Ser/Lys-“co-adaptation pocket” to inhibit lung cancer cell epithelial–mesenchymal transition

**DOI:** 10.18632/oncotarget.19599

**Published:** 2017-07-26

**Authors:** Xiao-Yu Ai, Heng Zhang, Shao-Yan Gao, Yuan Qin, Wei-Long Zhong, Ju Gu, Meng Li, Kai-Liang Qiao, Qin Tian, Zhan-Hong Cui, Jia-Huan Yang, Zhun Bi, Ting Xiao, Shuang Chen, Hui-Juan Liu, Hong-Gang Zhou, Tao Sun, Cheng Yang

**Affiliations:** ^1^ State Key Laboratory of Medicinal Chemical Biology and College of Pharmacy, Nankai University, Tianjin, China; ^2^ Tianjin Key Laboratory of Molecular Drug Research, Tianjin International Joint Academy of Biomedicine, Tianjin, China

**Keywords:** antitumor, EMT, co-adaptation pocket, ERK2, lung cancer

## Abstract

Sesquiterpene lactones (SL) have a wide range of applications in anti-tumor and anti-inflammatory therapy. However, the pharmacological mechanism of such substances is not clear. In this study, parthenolide (PTL) was used as an example to explore the anti-tumor effect of natural molecules and their common mechanism. We showed that PTL inhibited the proliferation and migration by reverse EMT via the ERK2/NF-κB/Snail pathway *in vivo* and *in vitro*. Interestingly, Multiple potential targets of PTL contain a Gly-Leu-Ser/Lys-“co-adaptation pocket”. This inspiring us analogies of PTL may also bind to these target proteins and play a similar function. Significantly, the Concept of co-adaptation pocket may help to increase the selectivity of drug research and development.

## INTRODUCTION

PTL is a natural antitumor agent extensively investigated and currently tested in clinical studies for leukemia and neurological tumors [1]. PTL induces specific mechanisms of anticancer and anti-inflammatory actions, including effects on the NF-κB pathway [2–4]. PTL is also used to treat inflammatory bowel disease, rheumatoid, and nephritis [5–7].However, PTL treatment for lung cancer and its pharmacological properties have yet to be reported.

In addition to the NF-κB pathway, the molecular mechanisms of PTL involve anti-oxygen free radicals and changes in microtubule functions, and they inhibit tumor growth; as such, PTL can be used in numerous applications in clinical settings [8, 9]. However, these molecular mechanisms, particularly anti-metastasis mechanisms, do not clearly explain the unique pharmacological properties of PTL. In our study, the effects of PTL on EMT, which is the core process of tumor metastasis [10–12], were investigated through *in vitro* and *in vivo* studies on cancer cell function, EMT biomarkers, and transition markers. Our results revealed that ERK2 was the target of PTL, and PTL could effectively suppress and reverse the EMT of tumors via the ERK2/NF-κB/Snail pathway. Some differences in the regulation of EMT by PTL were observed among lung cancers with different origin sites.

## RESULTS

### PTL reduces cell viability and inhibits the migration and invasion of human lung cancer cell lines

**Figure 1:**
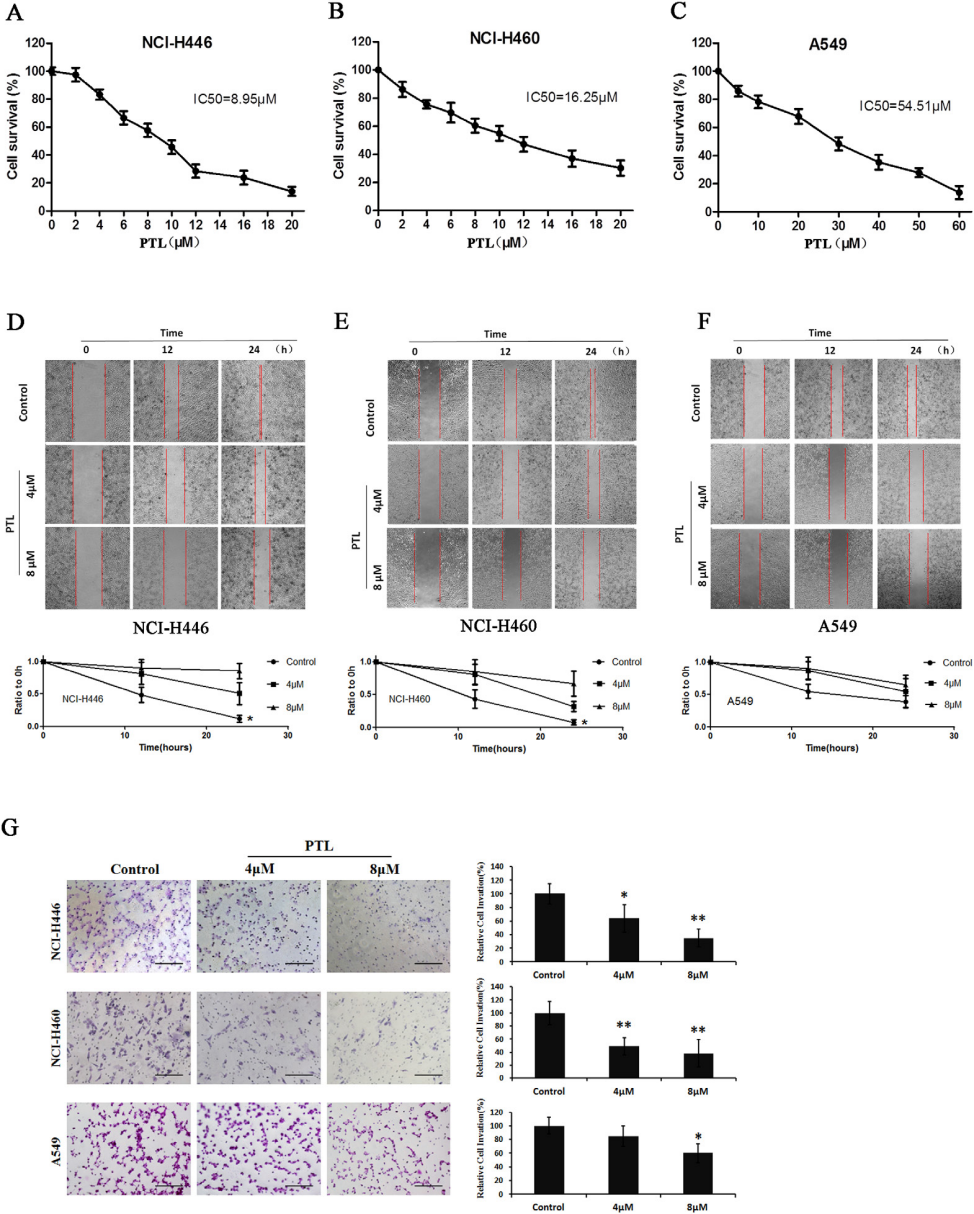
Effects of PTL on cell viability and migration **(A)** Survival of NCI-H446 cells treated with the indicated amounts of PTL for 48 h; IC_50_ = 8.95 µM. **(B)** Survival of NCI-H460 cells treated with the indicated amounts of PTL for 48 h; IC_50_ =16.25 µM. **(C)** Survival of A549 cells treated with the indicated amounts of PTL for 48 h; IC_50_ = 54.51 µM. **(D–F)** Cells were re-incubated in the medium containing 0, 4, and 8 µM PTL for 24 h. Results showed PTL inhibiting the NCI-H446 (Figure 1D), NCI-H460 (Figure 1E), and A549 (Figure 1F) cells. **(G)** Transwell chambers were utilized for the invasion assay, and images were obtained at 200× magnification. NCI-H446, NCI-H460, and A549 cells were treated with0, 4, and 8 µM PTL for 24 h. Each experiment was performed in triplicate. Results are the means of the three experiments, and the error bars represent standard deviation (^*^P ≤ 0.05, ^**^P≤ 0.01).

MTT assay was used to determine the effect of PTL on the viability of three different lung cancers with different origin sites, namely, human large cell lung cancer cell line NCI-H460, human small cell lung cancer cell line NCI-H446, and lung adencarcinoma cell line A549 (treated with PTL for 48 h). The molecular formula PTL was shown in Supplementary Figure 1. Figure 1A to 1C show that PTL significantly inhibited the proliferation of NCI-H446, NCI-H460, and A549 cells were in a dose-dependent manner. The human small cell lung cancer cell line NCI-H446, which originates from the neuroectoderm, was more sensitive to PTL with IC_50_ value of 8.95 µM than the two other lung cancer cell lines(16.25and 54.51µM). Wound healing assay was performed to assess the ability of PTL to inhibit the migration of the three lung cancer cell lines. In Figure 1D to 1F, PTL could suppress the migration of the three cell lines, and the strongest inhibitory effect was on NCI-H446. These results were in agreement with the results of MTT assay. To investigate whether PTL inhibits NCI-H446, NCI-H460, and A549 cell invasion, we utilized Matrigel-coated transwell chambers, and we found that PTL dose-dependently reduced the number of cell invasions through the Matrigel-coated filter in both cell lines compared with the control group (Figure 1G). Therefore, PTL markedly inhibited invasion in both three cell lines.

### PTL alters cellular morphologyin human lung cancer cell lines

**Figure 2:**
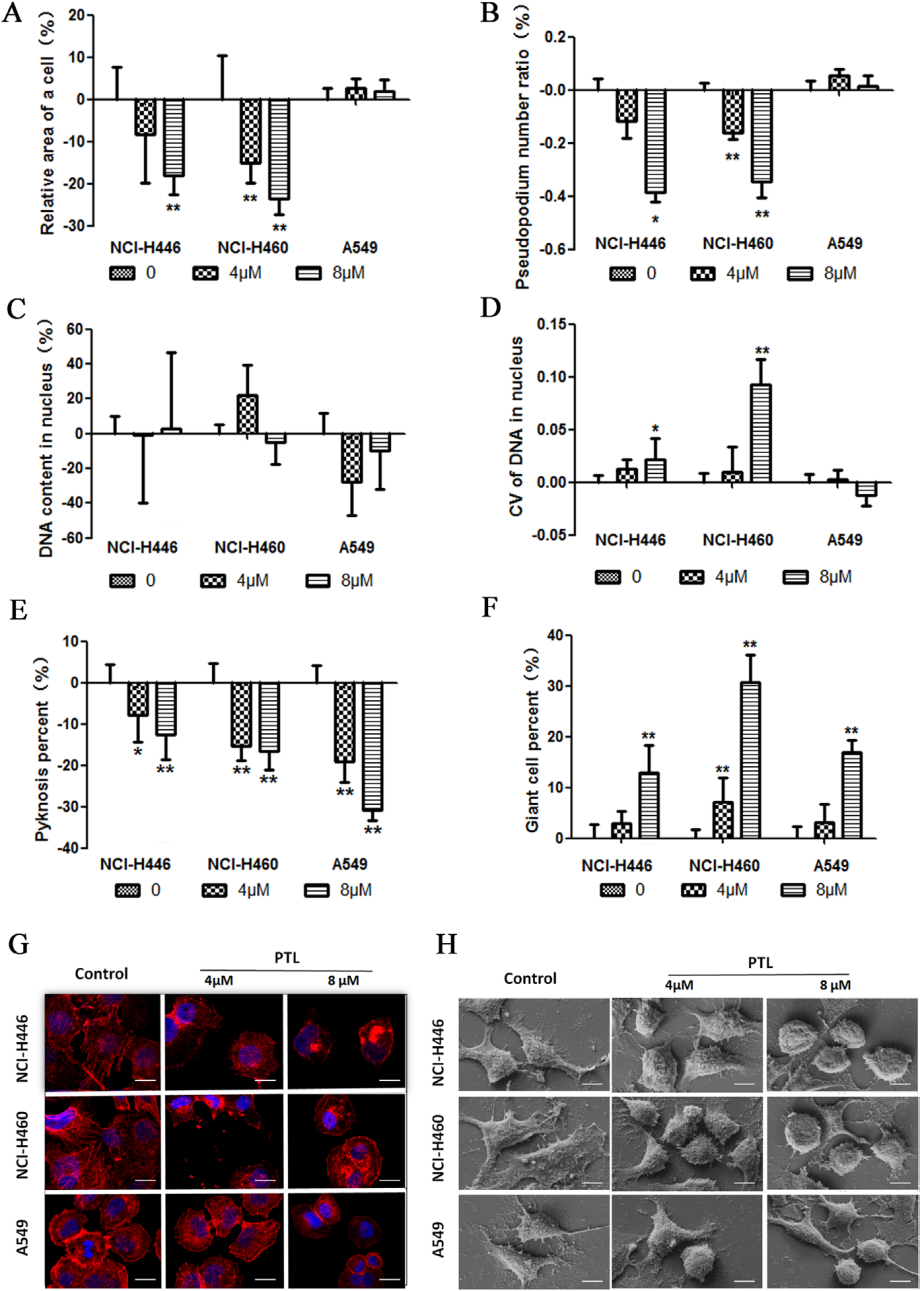
PTL alters cellular morphology **(A)** The cell area decreased in PTL-treated NCI-H446 and NCI-H460. No significant change was found in A549. **(B)** The pseudopodium number decreased in PTL-treated NCI-H446 and NCI-H460. No significant change was found in A549. **(C** and **D)** No significant change was found in DNA content treated with PTL, but CV of DNA in nucleus increased in NCI-H446 and NCI-H460. No significant change was found in A549. **(E)** The number of pyknosis decreased in all the three cell lines. **(F)** The number of giant cells increased in all the three cell lines. **(G** and **H)** Confocal images of actin cytoskeleton stained with rhodamine–phalloidin and scanning electron micrographs. Treatment of cancer cells with PTL led to theagglutination of β-actin, contraction of pseudopodia, cell shrinkage, and changes in cell shape (^*^P ≤ 0.05, ^**^P≤ 0.01).

Morphological features were characterized with HCS, a laser scanning confocal microscope and a scanning electron microscope. The results for NCI-H460 and NCI-H446 showed that PTL affected the cell area. The decrease in cell area implied a decrease in the cellular content. Simultaneously, PTL also decreased the number of pseudopodia and induced the formation of an irregular-sized nucleus with DNA contents that did not significantly differ (Figure 2C), but these changes were not significant in A549 (Figure 2A, 2B, and 2D). Karyopyknosis is an important sign of cell necrosis. However, for NCI-H460, NCI-H446, and A549, the karyopyknotic cell number decreased after PTL treatment, suggesting that PTL did not directly cause cell necrosis (Figure 2E). PTL induced an increase in giant tumor cells of the three cell lines in a dose-dependent manner, suggesting that PTL could induce cancer cell senescence and abnormal division (Figure 2F). In Figure 2G, cells were stained with a fluorochrome of F-actin. Low PTL doses induced the contraction of pseudopodia and altered the microfilament structure. By comparison, high PTL doses caused β-actin agglutination, cell shrink age, and changes in cell shape. The changes in cellular morphology in Figure 2H were similar to those observed in the cells shown in Figure 2G.

### PTL can inhibit and reverse the EMT biomarkers of lung cancer cells

**Figure 3:**
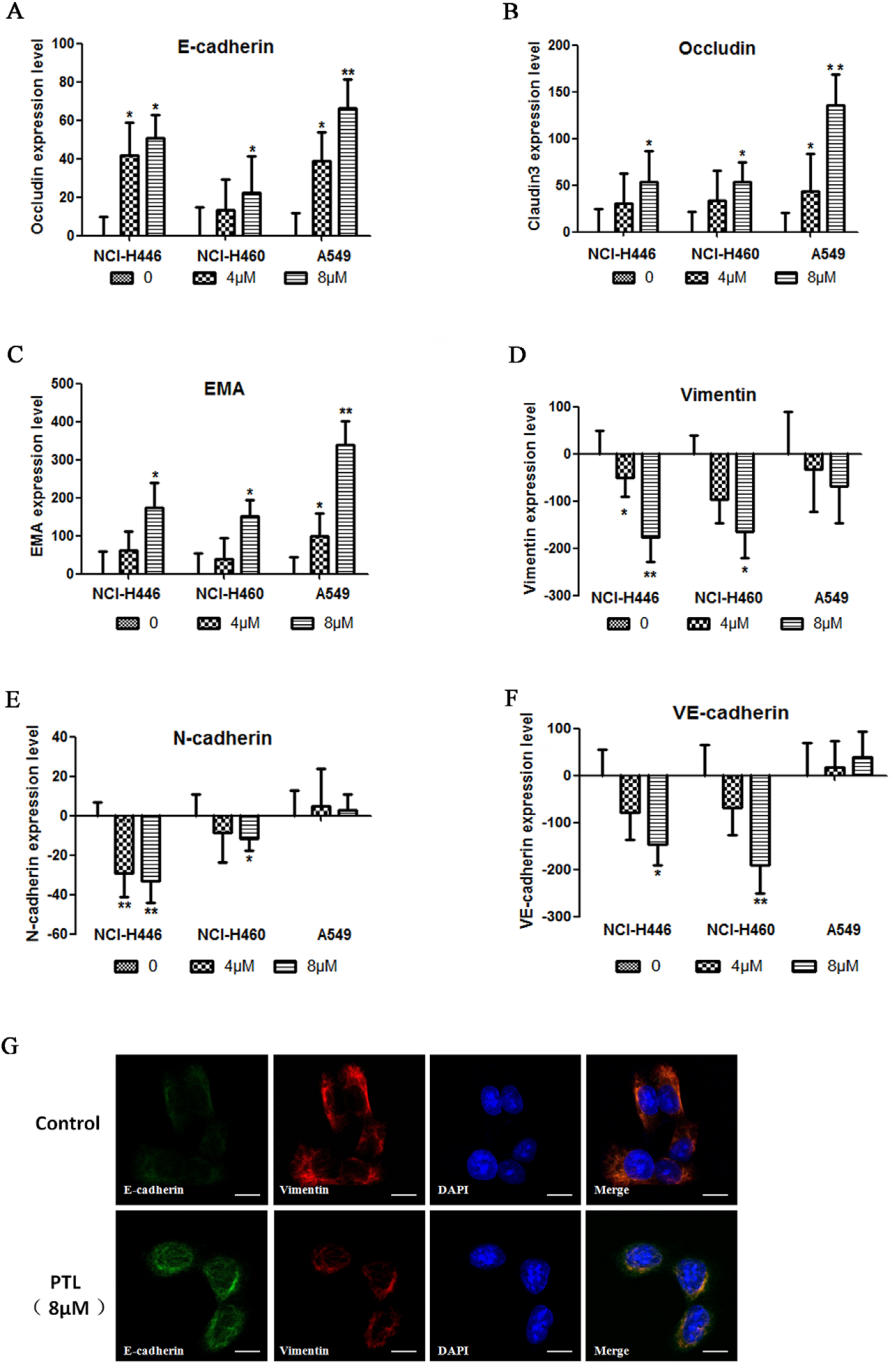
changes in EMT PTL reverses biomarkers **(A–C)** E-cadherin, Occludin, and EMA expression levels increased in a dose-dependent manner following PTL treatment. **(D–F)** In NCI-H446 and NCI-H460 cells, Vimentin, N-cadherin, and VE-cadherin expression levels decreased in a dose-dependent manner, whereas in A549, no significant change was found in these biomarkers following PTL treatment. **(G)** Typical images of immunofluorescent double staining for E-cadherin and Vimentin in NCI-H446 cells. Each experiment was performed in triplicate. The results are shown as the means of the three experiments, and the error bars represent the standard deviation (^*^P ≤ 0.05, ^**^P≤ 0.01).

The effect of PTL on the levels of epithelial and mesenchymal cell biomarkers by immunofluorescent staining in HCS systems was tested. In response to PTL treatment, epithelial cell biomarker (E-cadherin, Occludin, and EMA) expression increased in both cell lines in a dose-dependent manner (Figure 2A, 2B, and 2C). By contrast, mesenchymal cell biomarkers, such as Vimentin, N-cadherin, and VE-cadherin, the expression in NCI-H446 and NCI-H460decreased in a dose-dependent mannerin response to PTL treatment (Figure 2D, 2E, and 2F). No significant variation was observed in the expression of Vimentin, N-cadherin, and VE-cadherin in A549. These results showed that PTL could inhibit EMT in NCI-H446, NCI-H460, and A549, as well as reverse EMT in NCI-H446 and NCI-H460. However, PTL could not reverse EMT in A549. Immunofluorescent double staining was performed for E-cadherin and Vimentin and then visualized under a fluorescence microscope. Vimentin levels decreased and E-cadherin levels increased in NCI-H446 cells, and these results were similar to those of HCS (Figure 3G).

### Multiple potential targets of PTL contain a “co-adaptation pocket,” and ERK 2 was inhibited by PTL and thus induced EMT inhibition

**Figure 4:**
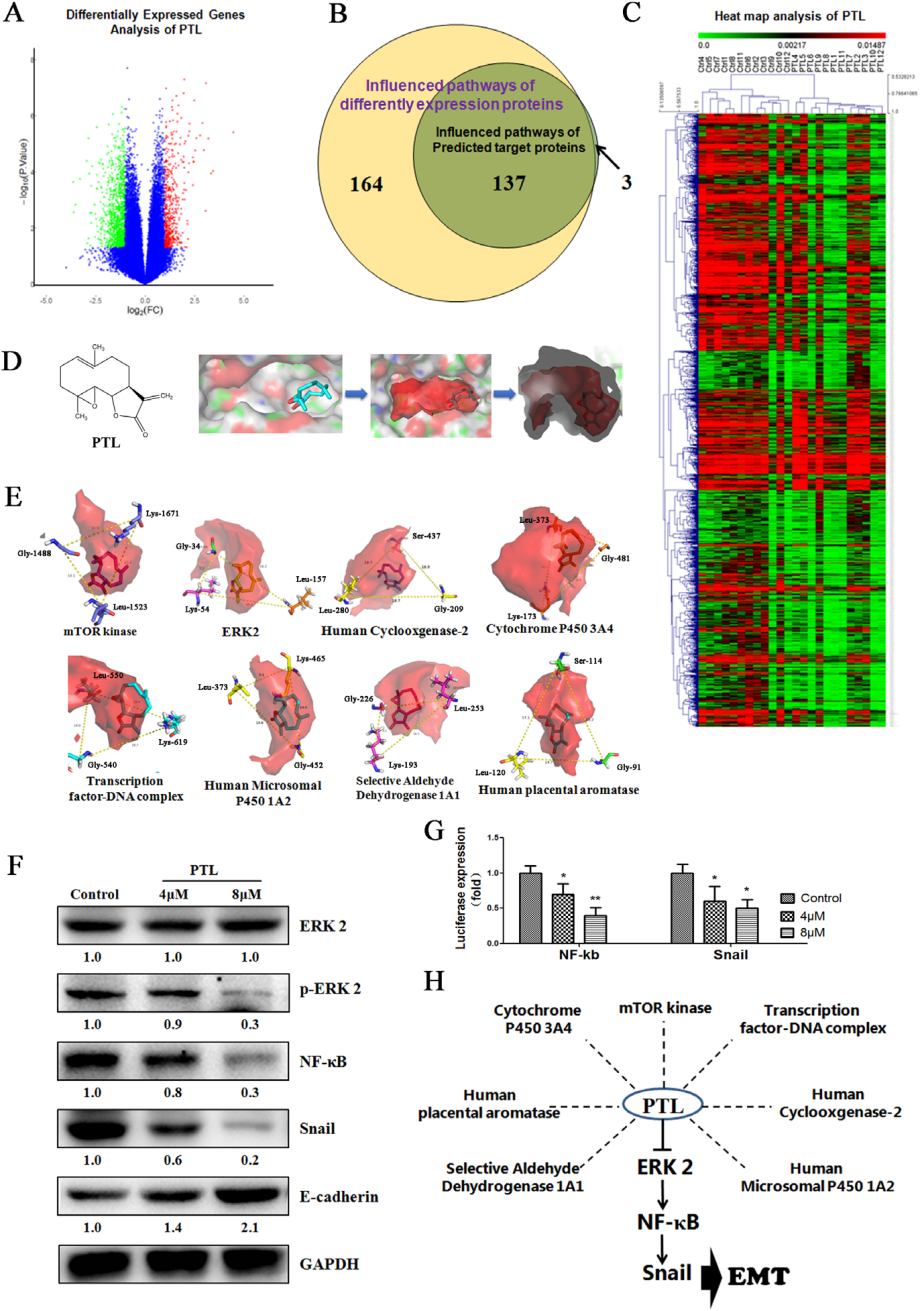
Targeting the Gly-Leu-Ser/Lys- “Co-adaptation pocket,” PTL suppresses EMT via ERK 2/NF-κB/Snail pathway **(A** and **C)** Gene expression microarray was used to interrogate the differentially expressed genes. **(B)** A total of 164 influenced pathways of the different expression proteins predicted from different expression genes, with which 137 of 140 were similar to the reversely predicted based PTL activity. **(D)** POVME 2.0 algorithm was used to measure and characterize the active pocket of proteins. **(E)**“Co-adaptation pocket” constitutes the Gly-Leu-Ser/Lys in the potential targets of PTL. **(F)** ERK 2 signaling was inhibited resulting in the suppression of EMT in NCI-H446. **(G)** Dualluciferase assay results suggest that PTL reduced NF-κB and Snail expression in NCI-H446 cell line. **(H)** Multiple potential targets of PTL have a “Co-adaptation pocket” and PTL inhibits ERK 2 resulting in EMT inhibition.

Gene expression microarray was performed to determine differentially expressed genes (Figure 4A and 4C). A total of 164 influenced pathways of these differential expression proteins were predicted. Moreover, 17 potential targets were calculated by reverse prediction based on PTL activity. Seventeen proteins consisted of 140 influenced pathways. Of these pathways, 137 were similar to differentially expressed proteins (Figure 4B).

POVME 2.0 algorithm measures and characterizes the active pocket of proteins (Figure 4D). There were 12 proteins with extremely similar active center structure (Supplementary Table 1). Based on the analysis of parthenolide interactions with target proteins, Gly, Lys, Leu, and Ser emerged around the active site of eight proteins at high frequency. We further measured the location relationship of those amino acids, and finally found amino acids that showed isosceles triangles in the active center, and the conformations and orientations of the parthenolide in the active center were identical in this triangle (Figure 4E). This characteristic is called “co-adaptation pocket.”

The score of molecular docking is shown in Table 1, in which ERK 2 was the best-rated protein. Thus, we detected the influence of PTL in ERK 2 by using NCI-H446 cell lines. The results showed no influence on ERK 2 expression, whereas in Figure 4F, the expression of p-ERK 2 treated with PTL was reduced. The activation of ERK 2 contributes to the regulation of NF-κB, and NF-κB and Snail, downstream of NF-κB, were reported to trigger the progression of EMT by activating different molecules. We analyzed the expression and activity of NF-κB and Snail using Western Blot and dual-luciferase assay. In Figure 4F and 4G, PTL could reduce the expression and activity of NF-κB and Snail. Our data indicated that ERK 2 signaling was inhibited, and the expression level of E-cadherin, which is the epithelial cell biomarker was increased when treated with PTL (Figure 4F). These results indicated that PTL could have multiple potential targets affecting various functions in cancer cells, and a “co-adaptation pocket” existed in this protein. In Figure 4H, ERK 2 was one of the potential targets of PTL, and PTL could suppress EMT through the ERK 2/NF-κB/Snail pathway.

**Table 1:** The molecular docking score of potential targets and PTL

Potential targets	Docking score
ERK2	-5.137
mTOR kinase	-5.130
Human Cyclooxgenase-2	-5.081
Human placental aromatase	-5.045
Cytochrome P450 3A4	-4.583
Transcription factor-DNA complex	-4.381
Human Microsomal P450 1A2	-3.512
Selective Aldehyde Dehydrogenase 1A1	-3.102

### PTL exhibits an antitumor effect on a mouse xenograft model

**Figure 5:**
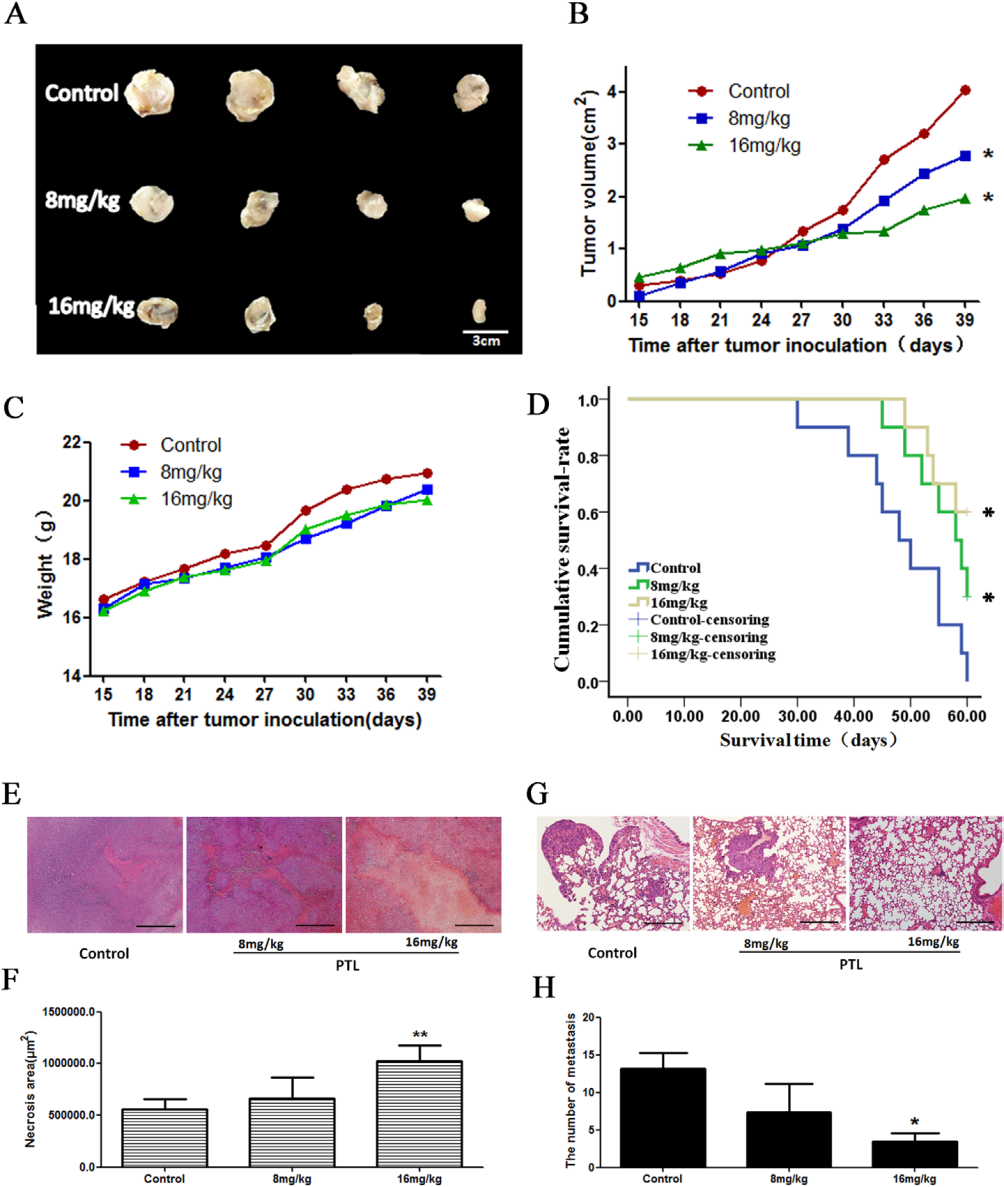
Effect of PTL on a mouse xenograft model Mice were treated with saline or PTL(8 and 16 mg/kg) for 25 d. **(A** and **B)** Changes in the tumor volume of LLC xenografts. PTL reduced the tumor volume. PTL treatment inhibited xenograft growth in a dose-dependent manner. **(C)** Body weights (g) of animals with LLC xenografts.**(D)** The median survival time was prolonged in a dose-dependent manner following PTL treatment. **(E** and **F)** The rate of necrotic regions of tumor markedly increased in tumor tissues of mice. **(G** and **H)** The number of tumors that shifted stoves markedly decreased in lungs of mice with LLC xenografts. Each experiment was performed in triplicate. Results show the means of the three experiments, and the error bars represent standard deviation (^*^P < 0.05 and ^**^P < 0.01).

We examined the effects of PTL on LLC xenografts in C57 BL/6 mice. PTL treatment inhibited the tumor growth in a dose-dependent manner with nearly no effect on body weight (Figure 5A to 5C). The median survival time of PTL groups increased compared with the control group (Figure 5D). The areas of tumor necrosis were increased when treated with PTL in a dose-dependent manner (Figure 5E and 5F). The number of tumors that shifted stoves markedly decreased in lungs of mice with LLC xenografts treated with PTL (Figure 5G and 5H).Overall, these results strongly suggested that PTL inhibited the tumor growth and metastasis.

### PTL alters EMT marker levels and inhibits the ERK2/NF-κB/Snail pathway in cancer tissues

**Figure 6:**
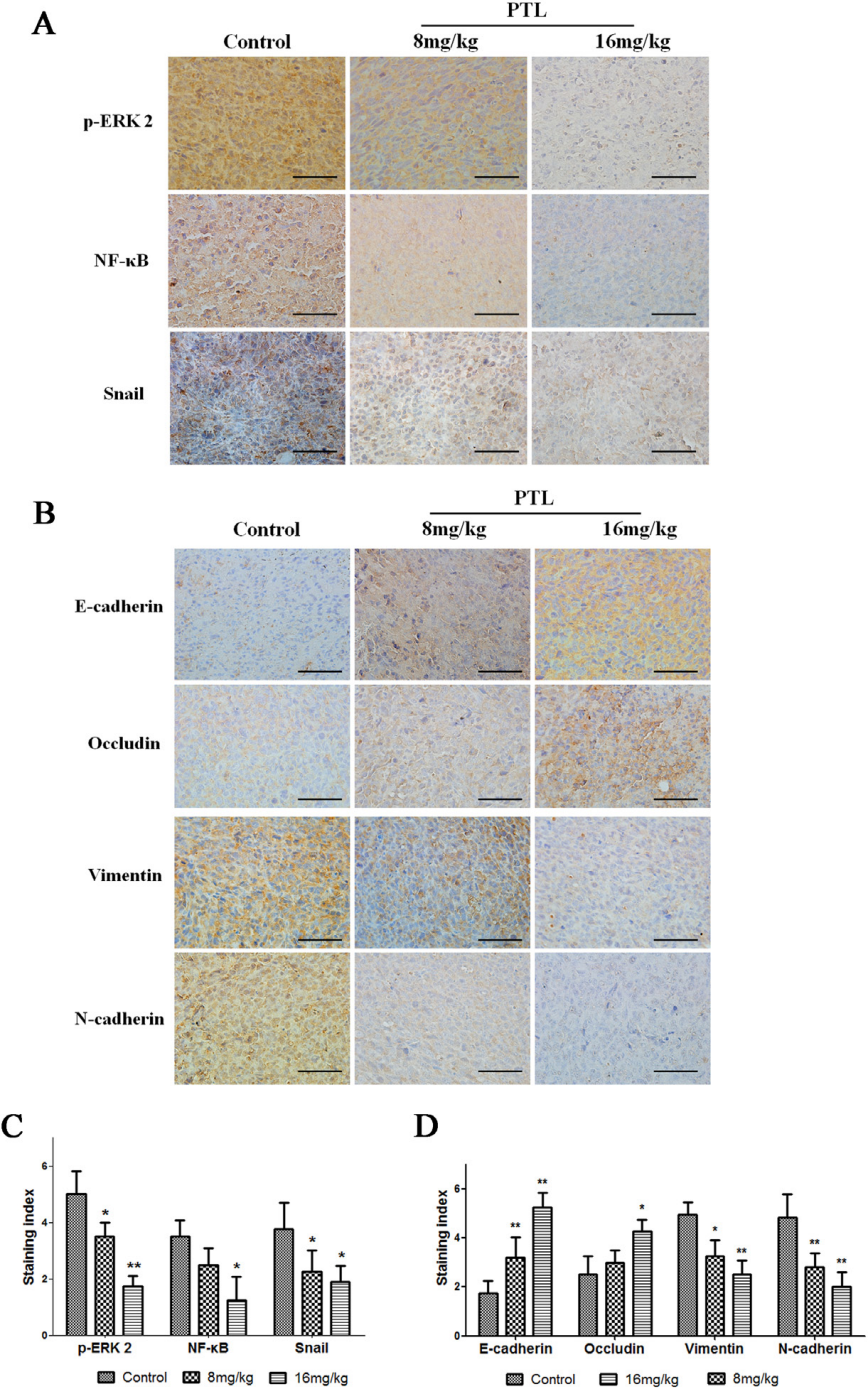
Effect of PTL on p-ERK 2, NF-κB, Snail, and EMT protein levels Brown or yellow staining was observed in the cytoplasmor nucleus. **(A** and **C)** Representative photographs of treated and untreated cells. PTL treatment reduced p-ERK 2, NF-κB, and Snail staining compared with sections obtained from control mice. **(B** and **D)** Representative photographs of treated and untreated cells. PTL treatment increased E-cadherin and Occludin staining and reduced Vimentin and N-cadherin staining compared with sections obtained from control mice. Each experiment was performed in triplicate. Results show the means of the three experiments, and the error bars represent standard deviation (^*^P < 0.05 and ^**^P < 0.01).

PTL treatment reduced immunohistochemical staining for ERK2, NF-κB, and Snail in a dose-dependent manner (Figure 6A and 6C). Immunohistochemical staining for E-cadherin, Occludin, Vimentin, and N-cadherin showed that PTL increased E-cadherin and Occludin levels, whereas it decreased Vimentin and N-cadherin levels in tumor tissues (Figure 6B and 6D). These results showed that PTL inhibited EMT through theERK2/NF-κB/Snail pathway in tumor tissues, which were in good agreement with cell experiments results.

## DISCUSSION

PTL is a natural molecule that shows excellent anti-inflammatory and antitumor activities [2, 13]. The first written records regarding the anti-inflammatory effect of PTL were provided in 1597 in Europe [1]. In 1973, PTL from *Magnolia grandiflora* was shown to exhibit antitumor properties for the first time [14].

PTL preferably inhibits tumors from nerve fibers and blood system [15, 16]. For instance, PTL prevents the growth of small cell lung cancer originating from the neuroectoderm. Our results showed that the strongest effect of PTL was found in NCI-H446, which is a small cell lung cancer cell line, followed by NCI-H460, which is a large cell cancer cell line. By comparison, A549, which is a type of lung adenocarcinoma, was not sensitive to PTL.NCI-H446 is a small cell lung cancer cell line, and NCI-H460 is a large cell cancer cell line. Both NCI-H446 and NCI-H460 were derived frompleural effusion and have more mesenchymal cell properties than A549 cells which are adenocarcinomic human alveolar basal epithelial cells. By comparison, A549was not sensitive toPTL. Therefore, cells with mesenchymal cell properties are more sensitive to PTL than malignant epithelial tumors. EMT is an accepted key process of tumor metastasis, in which cells originating from the epithelium acquire the properties of mesenchymal cells and thus causing metastasis and invasion [17–19]. PTL targets cancer cells, which change from an epithelial cell phenotype to a mesenchymal phenotype to inhibit and reverse EMT.

Our results also demonstrated that PTL could inhibit EMT in different lung cancer cells. An increase inepithelial biomarker sindicated that PTL could inhibit EMT in the three lung cancer cell lines.However, PTL could regulate the expression of mesenchymal biomarkers in NCI-H446 and NCI-H460 cell lines but could not affect A549 cell lines. These results suggested that PTL could inhibit and reverse EMT through complex patterns in certain cells.

Cell morphology analysis revealed that PTL influenced the cytoskeleton, pseudopodium, and cell nucleus. Moreover, different cell lines displayedvarious degrees of the influence. Their common feature was aged giant cells and abnormal mitotic giant cells caused by PTL. PTL is different from cytotoxic drugs because it does not cause tumor cell necrosis [20]. However, *in vivo* studies have shown that high-dose PTL creates a large necrotic area in tumor tissues. This necrotic area is indirectly related to tumor tissues but is related to ischemia caused by the inhibition of angiogenesis. In addition, PTL could inhibit the growth and metastasis of tumors *in vivo*. Immunohistochemical analysis results agreed with immunofluorescence results. Therefore, PTL could inhibit EMT.

PTL performs multiple activities, including anticancer and anti-inflammation. Various PTL targets, such as NF-κB,IKK, thioredoxin, and tubulin carboxypeptidase [21, 22]. Our results showed many potential targets, including ERK2. However, our predicted results showed that a “co-adaptation pocket” in the target proteins consisted of Gly-Leu-Ser/Lys in a triangular form.

ERK signaling pathway couples the obtained signals from cell surface receptors to transcriptional factors and regulates the activity of important proteins involved in proliferation, migration, cell cycle, and apoptosis through the major role of ERKs [23]. ERK1 and ERK2 are regulated differentially in response to specific extracellular stimulior cellular contexts [24]. ERK2 plays a larger part in tumor metastasis than in other processes [25, 26]. Irreversible ERK2 inhibitors, such as FR148083, inhibit their target proteins through the four hydrogen bonds (Met 108, Ser 153, Lys 114, and Asn 154) [27]. Our results showed that PTL inhibited ERK2 through the three other hydrogen bonds (Gly 34, Lys 54, and Leu 157).Thus, EMT was inhibited via the ERK 2/NF-κB/Snail pathway. On the one hand, most potential targets of PTL may contain a Gly-Leu-Ser/Lys-“co-adaptation pocket” in the active site. On the other hand, small molecules, which fit the “co-adaptation pocket,” may perform a similar function.

No report was found on PTL as an EMT inhibitor. Our study provided a basis for further clinical trialson PTL, which may be a potential EMT inhibitor via the ERK2/NF-κB/Snail pathway. Therefore, our study revealed insights into relevant small molecules and therapeutic targets for further research and development of antitumor drugs.

## MATERIALS AND METHODS

### Ethics statement

This study was approved by the Institutional Animal Care and Use Committee at Tianjin International Joint Academy of Biomedicine in accordance with national and international guidelines. All animals in this experiment were well taken care of.

### Materials

PTL was purchased from Aladdin (Shanghai, China). Crystal violet and 3-(4, 5-dimethylthiazol-2-y1)-2, 5-diphenyltetrazolium bromide (MTT) were purchased from Sangon Biotech (Shanghai, China). The antibodies to Occludin, Claudin3, EMA, N-cadherin, VEGFR1, CD34, and Vimentin were purchased from Affinity Bioreagents(Colorado, USA). The immunofluorescence staining kit with FITC-labeled goat anti-rabbit IgG and cytoskeleton red fluorescent probe Actin Red were purchased from KeyGENBioTECH (Nanjing, China). A dual luminescence assay kit was purchased from GeneCopoeia (Guangzhou, China).

### Cell culture

Human lung cancer cell lines, namely, NCI-H460, NCI-H446, and A549Lewis lung carcinoma (LLC) were obtained from KeyGen Biotech (Nanjing, China) and were cultured in Roswell Park Memorial Institute 1640 medium (Hyclone, USA) supplemented with 10% heat-inactivated (56 °C, 30 min) fetal calf serum (Hyclone, USA). The cell culture was maintained at 37 °C with 5% CO_2_ in a humidified atmosphere.

### Cell viability assay

MTT assay determines the cell viability. NCI-H460, NCI-H446, and A549 (5 × 10^3^cells/mL) were seeded in 96-well culture plates. After overnight incubation, cells were treated with various PTL concentrations. After 48-h incubation, cell viability was measured after the addition of 20 µL of MTT at 37 °C for 4 h. Subsequently, 150 µL of dimethyl sulfoxide was added to dissolve the formazan crystals. Optical density was determined at 570 nm with a microplate reader (Multiskan™ FC, Thermo Scientific, Waltham, MA, USA).

### Wound healing assay

NCI-H460, NCI-H446, and A549 cells were grown on a 35-mm dish plate to 100% confluence and then scratched to form a 100 µm wound using sterile pipette tips. Cells were then cultured both in the presence and absence of PTL for 24 h. Images of the cells that were taken using a light microscope (Nikon, Japan) were recorded at 12 and 24h.

### Immunofluorescent staining

NCI-H460, NCI-H446, and A549 cells (4 × 10^3^ cells/mL) were seeded in 96-well culture plates. After overnight incubation, NCI-H460, NCI-H446, and A549 cells were treated with various PTL concentrations. After 24-h incubation, cells were washed with PBS twice, fixed with 10% formalin in PBS, permeabilized, and blocked with PBS that contains NP-40 (0.1%) and bovine serum albumin (BSA, 3%). Then, cells were incubated in the same solution containing primary antibodies specific for Occludin antibody (1:50 dilution), Claudin3 antibody (1:100 dilution), EMA antibody (1:100 dilution), N-cadherin antibody (1:100 dilution), VEGFR1 antibody (1:100 dilution), CD34 antibody (1:100 dilution), or Vimentin antibody (1:100 dilution) for 1 h at room temperature(25 °C). Cells were washed with PBS four times and incubated with secondary antibody (1:200 dilution) for 30 min at room temperature. The cells were washed with PBS four times and then covered with Hoechst 33342 dye for 30 min at room temperature. Cells were washed with PBS four times. Proteins were visualized using high-content screening (HCS) systems.

### Cytoskeleton staining

NCI-H460, NCI-H446, and A549cells (5 × 10^4^ cells/mL) were seeded in 24-well culture plates on glass slides. After overnight incubation, cells were treated with various PTL concentrations. After a 24-h incubation, cells were washed with PBS twice, fixed with 10% formalin in PBS, permeabilized, and blocked with PBS that contains NP-40 (0.1%) and BSA (3%). The cells were then incubated in the same solution containing cytoskeleton red fluorescent probe Actin Red (1:40 dilution) for 20 min at room temperature(25 °C). Cells were washed with PBS twice before the glass slides with cells were placed on a microslide with DAPI-Fluoromount-G. Cells were visualized under a laser scanning confocal microscope (Nikon, Japan).

### Biacore assay

SPR experiments were performed using a Biacore 3000 instrument (GE Healthcare, Piscataway, NJ, USA). ERK2 was purified from NCI-H460(ERK2 or mutated ERK2 was over expression) cell lines using antibody-containing immunomagnetic beads. ERK2 was immobilized on CM5 sensor chips using the BiacoreAmini Coupling Kit according to the manufacturer’s instructions. PTL was diluted in running buffer and then injected into ERK2-immobilized CM5 sensor chips at concentrations of 0μM, 1.5625μM, 3.125μM, 6.25μM, 12.5μM, 25μM and 50μM or 0μM, 20μM, 40μM, 80μM, 160μM, 320μM. The surface of the control chip was prepared in the same manner and was used for data correction. Data analysis was performed using BIAevaluation software.

### Scanning electron microscopy

Cells grown on climbing films were treated with or without PTL. After 24h of incubation, the cells were fixed using ethyl alcohol, dried with a gradient concentration of tertiary butyl alcohol and finally coated with gold. Images of the cells were obtained using a scanning electron microscope (LEO 1530 VP, Germany).

### Dual-luciferase assay

Cells were transfected with dual-reporter constructs using transfection reagents. After changing into fresh medium 24 h after transfection, NCI-H446 and A549 cells were treated with various concentrations of doxycycline hydrochloride. After 48 h, the culture medium was collected into a 96-well white plate to measure luminescence with a luminometer.

### “Pocket” building

17 proteins with similar property were calculated by reverse prediction of the activity-based target of the parthenolide. Eight proteins with X-ray structure were downloaded from protein data bank (PDB), and others were obtained structures by SWISS-MODEL homologous modeling. 15 proteins with fit structures were selected for further study. Povme 2.0 algorithm was used to measure and characterize the pocket of proteins. Sphere of points was added to the pocket-encompassing region, centered on with an appropriate radius of each protein. Then, the shapes and characteristics of protein were generated. In order to obtain the three-dimensional structure of the protein active center, the active center of the protein was covered with the grid. 12 proteins with extremely similar action center structure, which were proves that the target reverse prediction was exactly.

Molecular docking was performed with parthenolide and 12 characteristics proteins. Active sites of proteins were used same as Povme pocket-encompassing region. Ligand and protein complex was saved in PDB format, and further analysis with Pymol software. 8Å residues around the ligand were selected and analysis the interactions of parthenolide and proteins.

### Animal studies

Five-week-old C57 BL/6 female mice were maintained in a specific pathogen-free animal care facility according to institutional guidelines. All animals in this experiment were well taken care of. LLC xenografts of tumors were established by subcutaneous injection of 1 × 10^7^ cells (suspended in PBS) into the flank. One day after tumor cell inoculation, the mice were randomly divided into three groups (*n* = 4). After the tumors reached an approximate volume of 100 mm^3^ (approximately six weeks after injection), mice in the treatment groups were treated with8 mg/kg or 16 mg/kg PTL through intraperitoneal injection, whereas mice in control groups were injected with saline. The body weight of each mouse was measured at different time points following tumor implantation. Tumor diameters were measured every day, and tumor volumes were calculated according to the following formula: V = ab2/2, where *a* is the length of tumor and *b* is the width of tumor. Thirty nine days after treatment, all mice were sacrificed after excessive anesthetization(60 mg/kg pentobarbital sodium) and xenografts and lungs were resected and measured.

Another 30 mice were allocated randomly to the three groups as described above (10 mice per group) to measure the survival rates. Each mouse was injected with1 × 106 cells (suspended in PBS) in the caudal vein. The survival time of each mouse was recorded.

### Immunohistochemical analysis

LLC xenografts of tumors were fixed in 4% paraformaldehyde, embedded in paraffin, cut into 4 µm-thick slices, and placed on slides. Tissues were deparaffinized using xylene, dehydrated in decreasing concentrations of ethanol, and incubated with 3% hydrogen peroxide for 15 min to block endogenous peroxidase activity. For the antigen retrieval, tissues were treated with citrate buffer saline (pH 6.0) for 15 min at 95 °C. Tissues were incubated with normal goat serum for 20 min at room temperature to block unspecific labeling. Tissues were then incubated with the corresponding primary antibodies in a humidified chamber overnight at 4 °C. Diaminobenzidine and hematoxylin were used for color development and as a counterstain, respectively. Primary antibodies and dilutions were as follows: Occludin antibody (Affinity, dilution 1:100), Claudin3 antibody (Affinity, dilution 1:100), EMA antibody(Affinity, dilution 1:50), N-cadherin antibody(Affinity, dilution 1:100), VEGFR1 antibody(Affinity, dilution 1:100), CD34 antibody(Affinity, dilution 1:200), and Vimentin antibody(Affinity, dilution 1:100). Tumor cells with brown cytoplasm or nucleus or membrane were considered positive and then scored based on four classes: none (0), weak brown (1+), moderate brown (2+), and strong brown (3+). The percentage of stained tumor cells was divided into five classes: 0 for negative cells, 1 for 1%–25%, 2 for 25%–50%, 3 for 50%–75%, and 4 for >75%.

### Statistical analyses

Data were expressed as means ± standard deviation. Comparisons between groups were performed through one-way ANOVA followed by Bonferroni’s post hoc test (SPSS version 17.0, SPSS Inc., Chicago, IL, USA). Significance level was set at P < 0.05.

## SUPPLEMENTARY MATERIALS FIGURE AND TABLE



## Abbreviations:

SL: sesquiterpene lactones
PTL: parthenolide
EMT: epithelial–mesenchymal transition
