# Supplementary material for: Sesquiterpene binding Gly-Leu-Ser/Lys-“co-adaptation pocket” to inhibit lung cancer cell epithelial–mesenchymal transition
Source: Oncotarget. 2017 Jul 26;8(41):70192–203. doi: 10.18632/oncotarget.19599 (PMC5642546; doi:10.18632/oncotarget.19599)
Supplement: Supplementary file 1 [file oncotarget-08-70192-s001.pdf]

Sesquiterpene binding Gly-Leu-Ser/Lys-"co-adaptation pocket" to inhibit lung cancer cell epithelial–mesenchymal transition

SUPPLEMENTARY MATERIALS

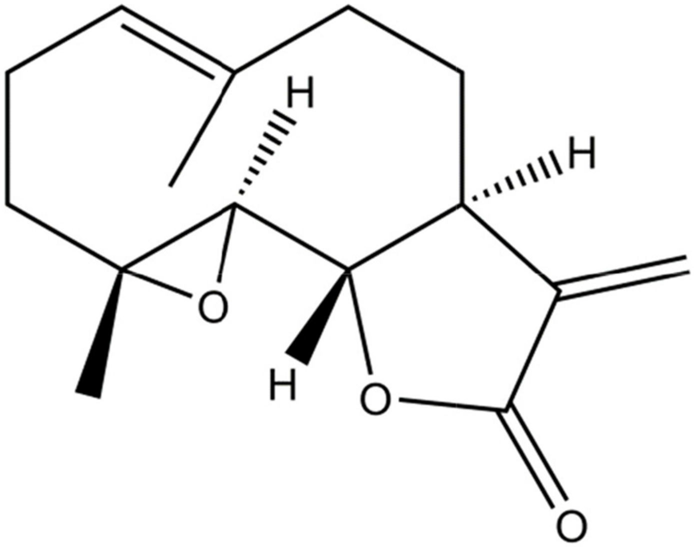

Supplementary Figure 1: The molecular formula PTL.

Supplementary Table 1: Potential targets calculated by reverse prediction based on PTL activity

Proteins	Property		
ERK	Potential targets calculated by reverse prediction based on PTL activity	Proteins with available structures	Proteins with extremely similar active center structures
Human Cyclooxgenase-2			
Human Microsomal P450 1A2			
Selective Aldehyde Dehydrogenase 1A1			
Cytochrome P450 3A4			
mTOR kinase			
Human placental aromatase			
Transcription factor-DNA complex			
thyroid stimulating hormone receptor			
Thrombopoietin			
Nitric oxide synthase,inducible			
Transcriptional activator Myb			
Microtubule-associated protein			
Nuclear factor NF-kappa-B p105 subunit			
Microphthalmia-associated transcription factor			
prelamin-A/C			
Anthrax lethal factor			
